# Omics-Based Approaches for the Characterization of Pompe Disease Metabolic Phenotypes

**DOI:** 10.3390/biology12091159

**Published:** 2023-08-23

**Authors:** Nuria Gómez-Cebrián, Elena Gras-Colomer, José Luis Poveda Andrés, Antonio Pineda-Lucena, Leonor Puchades-Carrasco

**Affiliations:** 1 Drug Discovery Unit, Instituto de Investigación Sanitaria La Fe, 46026 Valencia, Spain; 2 Pharmacy Department, Hospital Manises of Valencia, 46940 Valencia, Spain; 3 Pharmacy Department, Hospital Universitario y Politécnico La Fe, 46026 Valencia, Spain; 4 Molecular Therapeutics Program, Centro de Investigación Médica Aplicada, 31008 Pamplona, Spain

**Keywords:** lysosomal storage disorders, glycogen storage disease, Pompe disease, omics, multi-omics, metabolic phenotype

## Abstract

**Simple Summary:**

Pompe disease is produced by an enzymatic deficiency that leads to aberrant accumulation of glycogen in in multiple tissues, mainly muscle, causing progressive heart, respiratory and motor failure. Dysregulations observed in these patients are derived from glycogen accumulation but also to different secondary abnormalities. The characterization of the metabolic profile associated with this disease is a valuable approach to gain a larger view of all the metabolic dysregulations caused by the disease, and its potential correlation with clinical progression and response to therapies. This article describes the metabolic alterations reported to be significantly altered in Pompe disease patients in recent years. From a clinical perspective, this information could contribute to guide in the diagnosis, evaluation of disease severity, treatment decision and monitoring of Pompe disease patients.

**Abstract:**

Lysosomal storage disorders (LSDs) constitute a large group of rare, multisystemic, inherited disorders of metabolism, characterized by defects in lysosomal enzymes, accessory proteins, membrane transporters or trafficking proteins. Pompe disease (PD) is produced by mutations in the acid alpha-glucosidase (GAA) lysosomal enzyme. This enzymatic deficiency leads to the aberrant accumulation of glycogen in the lysosome. The onset of symptoms, including a variety of neurological and multiple-organ pathologies, can range from birth to adulthood, and disease severity can vary between individuals. Although very significant advances related to the development of new treatments, and also to the improvement of newborn screening programs and tools for a more accurate diagnosis and follow-up of patients, have occurred over recent years, there exists an unmet need for further understanding the molecular mechanisms underlying the progression of the disease. Also, the reason why currently available treatments lose effectiveness over time in some patients is not completely understood. In this scenario, characterization of the metabolic phenotype is a valuable approach to gain insights into the global impact of lysosomal dysfunction, and its potential correlation with clinical progression and response to therapies. These approaches represent a discovery tool for investigating disease-induced modifications in the complete metabolic profile, including large numbers of metabolites that are simultaneously analyzed, enabling the identification of novel potential biomarkers associated with these conditions. This review aims to highlight the most relevant findings of recently published omics-based studies with a particular focus on describing the clinical potential of the specific metabolic phenotypes associated to different subgroups of PD patients.

## 1. Introduction

Lysosomal storage diseases (LSDs) are a group of over 70 inherited metabolic disorders, frequently presented in childhood, caused by mutations in genes that affect the function of lysosomal hydrolases, accessory proteins, membrane transporters, or trafficking proteins, that finally result in the accumulation of biomolecules inside the lysosomes and lysosomal impairment. Although individually LSDs are defined as rare disorders, as a group they are relatively common, with an incidence as high as 21:100,000 live births in some countries [1,2,3]. From a genetic perspective, most LSDs are inherited as autosomal recessive, while only three have an X-linked inheritance pattern [4]. Traditionally, these disorders were grouped based on the accumulated biomolecule [5]; however, more recently, LSDs tend to combine this information with their molecular background and common pathophysiological mechanisms [6]. Among them, glycogen storage diseases (GSDs) are a subgroup of LSDs characterized by mutations in enzymes involved in glycogen metabolism, finally leading to the accumulation of glycogen in lysosomes [7]. GSDs are classified, based on the enzymatic deficiency, into different groups that can be further categorized in other subtypes [8]. Among the different subtypes, the GSD type II, also known as Pompe disease (PD), is one of the most studied [9]. The reported incidence of the disease varies in different populations, although it is estimated to range from 1:40,000 to 60,000 individuals [3,10,11,12].

PD is an autosomal recessive GSD caused by mutations on the encoding gene of the acid alpha-glucosidase (GAA) lysosomal enzyme, which is responsible for the hydrolysis of glycogen to glucose. This enzymatic deficiency leads to glycogen accumulation in various tissues, including musculoskeletal, cardiac, respiratory and nervous systems [13,14,15]. Diagnosis of PD includes accurate evaluation of clinical presentations, together with the measurement of GAA enzymatic activity, or protein abundance, in leukocytes, fibroblasts, urine, or rehydrated dried blood spots (DBS) for newborns [16,17,18,19,20] and molecular testing of the *GAA* gene [21]. In 2015, PD was added to the Recommended Uniform Screening Panel (RUSP) [16,22]. Since then, PD has been included in public newborn screening (NBS) programs in many countries, and has contributed to the identification of PD patients, even in asymptomatic cases [23,24,25,26].

From a clinical perspective, there are two main phenotypes described in PD: the infantile onset PD (IOPD) and the late onset PD (LOPD) phenotypes [27]. Moreover, patients with total enzymatic deficiency exhibit severe symptoms (e.g., hypertrophic cardiomyopathy, skeletal muscle myopathy) that finally lead to the patient’s death, while patients with a partial enzyme deficiency present less severe phenotypes [28,29]. Classic IOPD results from the complete or near-complete deficiency of GAA activity. This phenotype is clinically characterized by the onset of symptoms soon after birth [30], sometimes even manifesting prenatally [31,32], and requires early treatment for best outcomes [33,34,35,36]. In contrast, LOPD is characterized by later onset of symptoms, with a high heterogeneity in clinical presentation, usually characterized by muscle weakness with a limb girdle pattern, that leads to progressive respiratory insufficiency [37,38]. LOPD patients exhibit a variable spectrum of complex clinical phenotypes [39], ranging from an attenuate late-onset of disease with mild health symptoms to severe early-onset phenotypes that frequently result in patient death. Moreover, it is not clear whether these patients would benefit from prophylactic treatment to delay or prevent symptoms. Thus, there is no uniform consensus on the optimal time to initiate treatment in these patients [26,40].

Given the broad clinical variability and the unpredictability of different genotype–phenotype correlations in these patients, the application of omics technologies could remarkably contribute to improve the landscape of PD [41]. Indeed, the use of omics technologies (e.g., genomics, transcriptomics, proteomics and metabolomics) has greatly contributed to optimize the clinical management of different LSDs patients [6], and can provide new insights into the mechanisms underlying these disorders [42,43]. In addition, these technologies have also allowed the discovery of new genes involved in LSDs [44,45], and the identification of biomarkers associated with specific health conditions [46,47,48,49]. Notably, since these disorders are characterized by the accumulation of specific metabolites, the characterization of the metabolic phenotype of these patients can be used to guide in the diagnosis, evaluation of disease severity, treatment decision and monitoring of LSD patients [50]. In this context, since the metabolic composition can be influenced by the pathological processes of the disease or by the effect of specific treatments [51], the metabolic changes associated with these processes can be used to identify metabolic dysregulations related to the progression of these diseases, and to predict or monitor treatment response to therapies. Omics-based technologies have already proved to contribute to identify metabolic alterations associated to the pathophysiology of complex and heterogeneous diseases [52], such as irritable bowel syndrome [53] and colorectal cancer [54]. In PD patients, early diagnosis and early treatment have shown to be essential for a better outcome of patients [55,56,57,58]. Also, different factors (e. g., age at start of treatment, CRIM (cross-reactive immune material) status, extra lysosomal glycogen accumulation in muscle) are thought to be correlated with the high variability of response to ERT in PD patients [14,35,59,60,61,62]. Additionally, there is a current need to develop newer and more sensitive biomarkers related to disease burden, disease progression, optimal time to initiate ERT and response to current treatments [26,63,64,65,66,67]. Thus, metabolic phenotyping represents a powerful and promising approach for the characterization of clinically relevant PD metabolic phenotypes that could be translated to therapeutic benefits for these patients.

**Figure 1:**
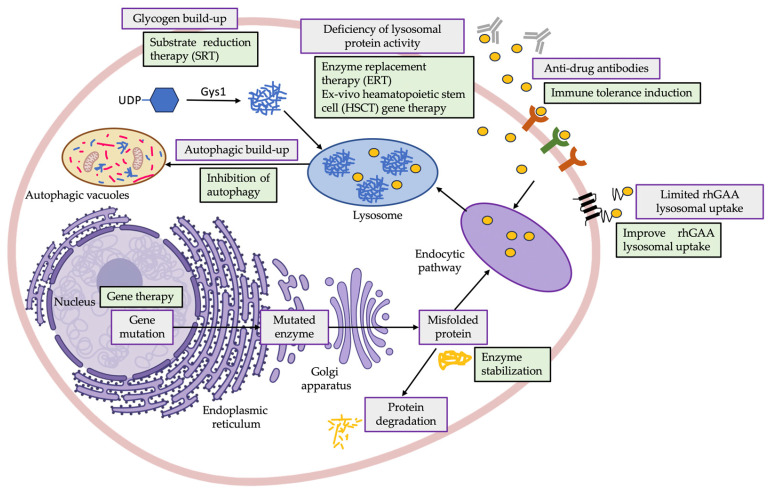
Graphical representation of the most common dysregulations observed in PD patients, derived from glycogen accumulation but also due to secondary abnormalities (e.g., impaired autophagy, activation of inflammation), and newer therapeutic approaches under investigation directed to restore lysosomal functionality and improve response to therapy in PD patients, including ERT (e.g., Myozyme) [69], enzyme stabilization (e.g., Cipaglucosidase alfa plus miglustat) [79], improving rhGAA lysosomal uptake (e.g., Avalglucosidase, Reveglucosidase, anti-CD63-GAA, Clenbuterol) [75,76,77], gene therapy (e.g., SPK-3006, ACTUS-101, AT845) [78], SRT (e.g., MZE001) [80], ex vivo HSCT gene therapy [81], immune tolerance induction (e.g., Methotrexate, Rituximab) [74,82] and inhibition of autophagy (e.g., AAV-mediated TSC knockdown) [83,84]. Created with BioRender. AAV-mediated TSC: adeno-associated virus-mediated tuberous sclerosis complex; ERT: enzyme replacement therapy; HSCT: hematopoietic stem cell; rhGAA: recombinant human acid alpha-glucosidase; SRT: substrate reduction therapy.

Regarding PD treatment, enzyme replacement therapy (ERT), given as recombinant human GAA (rhGAA), has been available for more than 15 years [68]. Although alglucosidase alfa (Myozyme/Lumizyme^®^, Sanofi-Genzyme, Cambridge, MA, USA) approval by the Food and Drug Administration (FDA) and the European Medical Agency (EMA) in 2006 completely changed the landscape for PD patients [69], ERT is currently the only available treatment option [59,70,71]. Moreover, this therapeutic strategy presents some limitations [64], including variability in its effectiveness among patients [9,59], limited bioavailability in the central nervous system (CNS) [72] and development of a strong immunologic response to treatment in some patients, that lead to less effective and sustained response to treatment [73,74]. Notably, efforts have been made in recent years towards the development of novel ERTs with improved lysosomal uptake (e.g., Avalglucosidase, Reveglucosidase, anti-CD63-GAA, Clenbuterol) [75,76,77], gene-based therapies (e.g., SPK-3006, ACTUS-101, AT845) [78] and also novel therapies targeting other disease-related mechanisms, including autophagy, immune response and others (Figure 1). In this scenario, identification of molecular markers that could contribute to evaluate the therapeutic efficiency would also be greatly beneficial for the development of these novel treatments.

Although not specific, urinary glucose tetrasaccharide (Glc4) seems to be the most important biomarker in measuring the progress of PD [85,86,87,88] and monitoring the therapeutic response to ERT [89,90]. For this reason, a number of studies have been performed to develop robust procedures based on mass spectrometry methods for the analysis of Glc4 in different biological samples [91,92,93,94] and to yield more evidence of its utility as a biomarker [91,95,96,97]. Other studies have emerged looking for new metabolic biomarkers to improve the clinical value of Glc4 for the diagnosis and prognosis of PD (reviewed in [85,98]). This review summarizes the results obtained in recent omics-based studies focused on the characterization of distinct PD metabolic phenotypes reported in PD patients, and also related with different clinical outcomes/treatment response in these patients. Metabolic phenotyping is a systems biology approach that seeks to comprehensively assess the metabolic status of an individual from a holistic perspective, based on the analysis of a multitude of biochemical components and not in the individual measurement of specific metabolites. These approaches are very valuable as they provide a deeper knowledge of the molecular mechanisms underlying the pathology. In the last years, the percentage of studies applying omics-based approaches for the characterization of the metabolic phenotype associated to PD has significantly increased. For this reason, this review has focused on studies published in the last five years. Further details on the specific criteria followed for the selection of the studies included in this review are included in the Supplementary Materials section (Figure S1). Out of the nine studies finally included in the review, four of them included data related to the identification of PD diagnostic biomarkers, five of them focused on the characterization of distinct PD phenotypes and four of them also evaluated the metabolic changes associated with the response to treatments in these patients. The following sections of this work describe the most relevant findings reported in these studies in relation to the pathophysiology of PD.

## 2. Omics Studies Directed to the Identification of Metabolic Pompe Disease Diagnostic Biomarkers

The detection of metabolic alterations associated with the development of PD may contribute to the identification of new diagnostic biomarkers and improve the diagnosis of this disease. In this context, a range of studies have compared the metabolic profile of PD patients and healthy individuals using different omics approaches aiming at the identification of potential metabolic biomarkers that could be clinically useful in PD diagnosis (Table 1). In these studies, mass spectroscopy (MS) was the analytical platform used for the identification of metabolic alterations in PD patients, and urine as the preferable sample type for analysis.

In the study conducted by Sidorina et al., data from proteomic and lipidomic analyses were combined to identify novel PD biomarkers and gain knowledge in the physiopathological mechanisms underlying PD [99]. Comparison of the proteomic profile of plasma samples from control subjects and PD patients showed significantly increased levels of two proteins involved in glucose metabolism, lactate dehydrogenase (LDHB) and pyruvate kinase (PKM), in these patients. Moreover, the pathway enrichment analysis performed in this study revealed that these changes could be related to alterations in the glucagon signaling pathway, connected with the breakdown of cell glycogen, suggesting an impairment of glucose/glycogen metabolism in these patients [102]. Also, given that LDHB is expressed in heart tissues and that increased levels of LDHB have been associated with heart damage [103], the authors suggest that this change could be a reflection of cardiac abnormalities observed in PD patients. Significantly lower levels of proteins related to phosphatidylcholine metabolism, glycosylphosphatidylinositol specific phospholipase D1 (GPLD1) and paraoxonase 1 (PON1) were also reported in PD patients in this study. Since PON1 regulates the hydrolysis of some phosphatidylcholines into lysophosphatidylcholines [104], down-regulation of this enzyme could explain the accumulation of phosphatidylcholines and reduction of lysophosphatidylcholines levels in plasma, which were also observed in the PD samples analyzed in this study. The results from this study highlight the potential of integrated multi-omics analyses for the identification of novel biomarkers but also for a better understanding of disease-related alterations.

Other studies have been focused on evaluating the performance of different analytical methods as metabolic screening tools for different LSDs causing an accumulation of oligosaccharides (OS) [105,106,107]. In this line, Semeraro and colleagues evaluated the performance of an ultra-high performance liquid chromatography mass spectrometry (UHPLC-MS/MS) which allowed to characterize the different oligosaccharide species in urine and dried urine spot (DUS) samples in a chromatographic run less than 30 min [94]. Glc4, a characteristic glycogen-derived tetrasaccharide in PD, is a known biomarker for PD [108] and for monitoring the response to ERT in these patients [89]. In this study, Glc4 and its isomer maltotetraose (M4) were the most significantly elevated OS found in PD patients’ samples. Interestingly, the authors found that this analytical method was able to detect increased concentrations of Glc4 in the urine and DUS in PD patients, but also in patients diagnosed with autophagy disorders coursing with cardiac abnormalities related to glycogen storage, such as Vici syndrome, Yunis–Varon syndrome and Danon disease patients.

Another study conducted by de Moraes and coworkers [100], which included a larger cohort of PD patients, also detected Glc4 as the most discriminative biomarker when comparing the control group with both LOPD and IOPD patients. This result was also in accordance with previous publications [86,109], therefore validating their analytical method. In addition to Glc4, the targeted analysis also revealed increased levels of other OS species, including hexose oligomers Hex5, Hex6, and Hex7. Moreover, the authors performed an untargeted strategy to identify additional metabolic alterations with diagnostic utility in PD patients. This allowed them to identify seven metabolites strongly associated with PD, showing significant statistical differences between healthy individuals and PD patients. Particularly, compared to control individuals, the metabolic profile of PD patients was characterized by higher Glc4, creatine, sorbitol/mannitol, L-phenylalanine, N-acetyl-L-aspartic acid and lower N-acetyl-4-aminobutanal and 2-aminobenzoic acid levels. Notably, although all candidate biomarkers showed area under the curve (AUC) values above 0.70, when the levels of these seven metabolites were combined for a multivariate ROC curve analysis, that metabolic panel showed superior discriminative capability (AUC > 0.96) compared to each individual metabolite.

Alterations in the urinary levels of Hex7 and Hex6, in addition to Glc4, were also found in PD patients in a study conducted by Hagemeijer et al. [101]. The individual analysis of the predictive potential of these metabolites revealed AUC values above 0.97. Hex6 showed the lower accuracy, while Hex7 exhibited slightly higher specificity and sensitivity when compared to Glc4, suggesting the potential value of Hex7 as a novel biomarker for the diagnosis of PD.

## 3. Omics Studies for the Characterization of Specific Pompe Disease Metabolic Phenotypes

Other studies have focused on identifying metabolic changes related to a particular disease phenotype (Table 2). In PD patients, glycogen accumulation due to the complete or near-complete deficiency of the GAA lysosomal enzyme mainly affects cardiac and skeletal muscles [9]. Thus, different studies aimed at exploring the metabolic profile of muscle biopsies from more and less severe PD phenotypes to gain deeper knowledge in the molecular processes underlying this disease. The majority of these studies relied on the analysis of tissue samples for characterizing the metabolic dysregulations specifically associated with disease severity, while the urine metabolic profile was explored in only one of these studies for the comparison of infantile-onset vs late-onset PD [100]. Regarding the strategy used for the identification of metabolic alterations, MS was the most widely used analytical platform, though the transcriptomic and the proteomic profiles were analyzed in one study each [110,111].

One of the hallmarks of PD is muscle atrophy [113,114,115,116] that occurs following a shift in protein synthesis and degradation towards protein degradation [117,118]. Hence, Lim and coworkers designed a metabolomics strategy in order to characterize how GAA knockout (GAA-KO) in the muscle affected the muscle metabolic profile in an animal model [84]. In this analysis, significantly elevated levels of total amino acids, particularly histidine, lysine, threonine, alanine, aspartate, glutamine and serine, were observed in the muscle of GAA-KO animals compared to the wild-type group. These changes were accompanied by an increased proteasome content and activity in the skeletal muscle of GAA-KO mice. These results are in accordance with the increase in both protein synthesis and degradation in PD patients reported in previous studies [117,118] that set the basis of the high protein and exercise therapy for PD patients [119,120,121].

The metabolic profiles of skeletal muscle from GAA-KO and wild-type mice were also compared in a more recent study conducted by Meena and colleagues [121]. In particular, GAA-deficient mice showed a metabolic shift from glucose towards fatty acid metabolism as the main energy source, characterized by lower levels of glycolytic metabolites and increased concentrations of glycogen synthesis precursors, as well as an increase in acetyl-CoA, tricarboxylic acid (TCA) cycle intermediates and carnitine levels. The authors suggested that these findings would be consistent with the shortage of glucose in these tissues, indicating lysosomal glycogen accumulation as well as dysregulation of recycled glucose would be involved in muscle damage in PD patients.

Other studies have reported different findings in relation to glucose metabolism observed at the transcriptome level. In the study conducted by Kinton et al., the transcriptomic profiles from healthy individuals’ and LOPD patients’ (ranging from 19 to 78 years age) muscle biopsies were compared to identify cellular processes that were specifically altered in LOPD patients [110]. The results obtained following a co-expression and pathway enrichment analysis revealed that, compared to healthy controls, LOPD patients showed enrichment in basic lysosomal function and biogenesis pathways, together with an increase in glycolysis and lipid-related metabolic process, such us sphingolipid and phospholipid metabolism. On the other hand, LOPD patients showed a significant attenuation in the mitophagy pathway and a disruption of calcium homeostasis, indicated by the increased enrichment in several pathways related to calcium ion transport, signaling and binding in these patients. Previous studies have also reported defects in lysosomal morphology and impaired lysosomal functions [9,122,123,124], alteration in glycolysis and lipid metabolism [99], and dysregulation of calcium homeostasis [125] in PD. Also, alterations in mitochondrial morphology, function and clearance [122,123,126,127] have been previously reported in relation to PD. Notably, in this study the authors observed similar changes in these pathways when analyzing a transcriptomic dataset from an external IOPD cohort of patients. This analysis indicated an enrichment of lysosomal function, glycolysis and lipid metabolism in IOPD patients compared to healthy controls. Nonetheless, impaired mitophagy was not observed in IOPD patients, indicating that dysregulation of this biological mechanism is particularly associated with the LOPD phenotype.

Increased glycolysis was also observed using a proteomics approach in a study conducted by Moriggi and coworkers focused on gaining deeper insights into the molecular players involved in the impairment of muscle metabolism of LOPD patients [111]. Using a double proteomic approach, based on two-dimensional difference gel electrophoresis (2D-DIGE) and label free liquid chromatography-mass spectrometry (LC-MS/MS) proteomics, the authors found altered levels of 178 proteins when comparing the proteomic profile of muscle biopsies from LOPD (ranging from 46 to 75 years age) and healthy subjects. Additionally, ingenuity pathway analysis revealed that, compared to healthy individuals, LOPD patients exhibited enhanced glycolysis and inhibition of oxidative phosphorylation (OXPHOS) suggesting that, in LOPD muscle biopsies, mitochondria are unable to oxidize substrates for ATP production, which is needed for muscle contraction.

A recent metabolomics study was focused on characterizing differences in the OS profile of IOPD and LOPD patients. The results from this study revealed that the urinary OS profile of IOPD patients was characterized by significant increased concentrations of Glc4 Hex5, Hex6, and Hex7, when compared to LOPD patients [100]. Notably, elevated Glc4 levels have previously been reported in IOPD [109].

## 4. Omics Studies for the Characterization of the Metabolic Response to Therapeutic Interventions in Pompe Disease

Over the last years, several studies have focused on exploring metabolic changes associated with the response to ERT or gene therapy (Table 3) in PD. Muscle biopsies were analyzed in all these studies, with one of them analyzing spinal cord samples [128]. From an omics perspective, the transcriptomic profile was characterized in two of the studies [110,128], while proteomics [111] and metabolomics [121] analyses were conducted in one study each. Regarding therapeutic intervention, ERT was administrated in three studies [110,111,121], whereas the effect of adeno-associated virus (AAV) vectors was evaluated in one study [128].

Although ERT is the standard of care for PD patients, many patients present poor outcomes due to the heterogeneous response and limited efficacy of the drug in clearing muscle glycogen storage [129]. Late-onset patients benefit from ERT for the first couple of years, but the effect of this therapy worsens through time, affecting muscle function [15,130,131]. Different studies have tried to elucidate the reason for these changes [132]. Nevertheless, it has been difficult to evaluate due to the clinical heterogeneity and the small number of samples that have been included in these studies. Following this purpose, a proteomic study conducted by Moriggi et al. compared the profile of the muscle proteome of LOPD before and after one year of ERT. The aim of the study was to explore the treatment’s effect on muscle tissues and to understand why ERT efficacy decreases over time in these patients [111]. Pathway enrichment analysis of significantly dysregulated proteins revealed a significant inhibition of glycolysis and gluconeogenesis following ERT. Based on these results, the authors suggested that these could be metabolic pathways specifically targeted by ERT. In addition, the authors reported that protein homeostasis was still not completely balanced even after one year of ERT, indicating that longer treatment times may be required to recover or maintain muscle function in these patients [133].

Metabolic alterations after ERT were also analyzed by Kinton and coworkers to investigate the early response of LOPD patients to treatment [110]. In this case, the analysis was performed for the comparison of the transcriptomic profiles of LOPD muscle biopsies before and after six months of ERT. Based on the observed transcriptomic changes, the authors did not report significant changes in glycolysis at this point of treatment. Nonetheless, other metabolic pathways were already partially attenuated or enhanced after the therapeutic intervention and were more similar to the control group. Particularly, calcium homeostasis, and sphingolipid and phospholipid metabolism of treated LOPD patients showed trends towards normalization or significant reductions in enrichment after ERT. In addition, mitophagy was significantly enriched after ERT, suggesting a potential complete recovery of mitochondria function and morphology after 6 months of therapeutic intervention.

Meena et al. also evaluated the potential of ERT [121], based on recombinant human GAA co-administrated with miglustat [134], to reverse the metabolic defects caused by lysosomal glycogen accumulation and lysosomal dysfunction in the muscle tissue of a GAA-KO mice model. In particular, following ERT, the metabolic profile was more closely related to the wild-type phenotype. This metabolic shift was mainly reflected by increased levels of glycolytic-related metabolites and lower concentrations of TCA cycle intermediates in the ERT-treated GAA-KO animals compared to untreated GAA-KO mice. Although the metabolic changes in glucose metabolism reported in this study differ from the changes observed in other studies based on the analysis of LOPD patients’ samples, the results from this study indicated that ERT either reversed or improved the metabolic alterations associated by a deficiency of GAA activity in this animal model [64].

A similar animal model was used in a different study for the evaluation of the efficacy of AAV in restoring GAA enzymatic activity and reverting the gene expression dysregulations observed in the skeletal muscle and spinal cord tissues of GAA-KO mice [128]. Comparing untreated GAA-KO mouse models with mice receiving four months of AAV therapy, Colella and colleagues reported significant restoration of GAA activity in both muscle and spinal cord biopsies, resulting in normalization of glycogen storage and mitophagy capacities. In addition, based on RNA-sequencing data analyses, the authors observed that the expression of over 90% of the genes found to be dysregulated in the skeletal muscle of untreated GAA-KO mice was either fully (65.7%) or partially (24.8%) normalized following AAV administration. Particularly, *GAA* gene transfer by AAV in GAA-KO mice restored expression levels of genes involved in bioenergetics and metabolism processes, including glycogen degradation, glucose and glucose-1-phosphate degradation, and serine and glycine biosynthesis pathways. The fact that the enhancement in glucose metabolism observed in this study is opposite to the results reported more recently by Moriggi et al. [111] could be attributed either to the technological platform used for the analysis or to the therapeutic intervention being evaluated (i.e., ERT vs. AVV). Regarding the transcriptomics profile of spinal cord samples, the expression of approximately 63% of the genes, which were mainly associated with pathways involved in nervous system disease, neuroinflammation, immunity and energy sensing, were fully or partially recovered after GAA restoration by AAV gene therapy. These results suggested that gene therapy based on AAV vectors could contribute to efficiently restore GAA activity in the muscle and nervous system, leading to restoration of the biochemical and transcriptomic defects observed in this GAA-KO animal model.

Overall, the results from these studies reflect that, following therapeutic interventions aimed at restoring GAA enzymatic activity, certain metabolic changes revert to the state observed in healthy individuals, while other metabolic changes are not affected after treatment. These results are in accordance with previous studies that have demonstrated that although ERT can improve clinical outcomes and survival of PD patients, it does not revert many other biological processes underlying this disease. In this line, several factors have been shown to contribute to skeletal muscle resistance to ERT, including defective autophagy [135], which is also associated with muscle atrophy [113,115,136].

## 5. Conclusions

**Figure 2:**
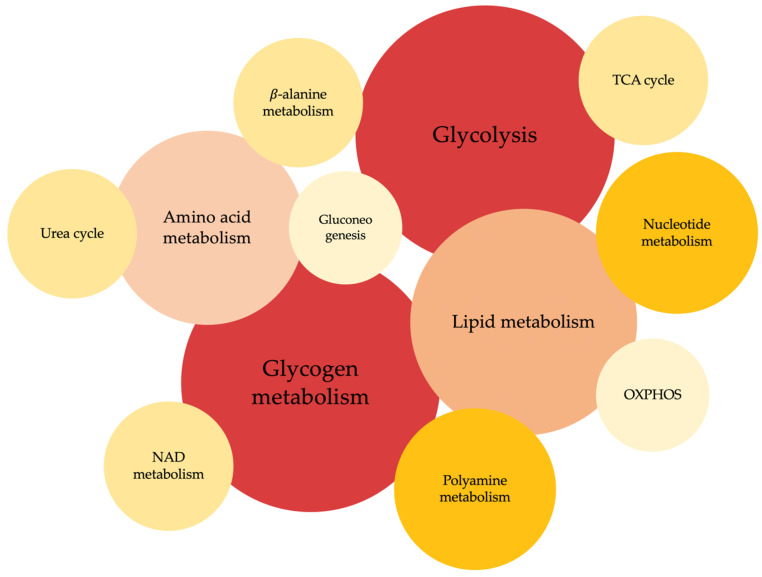
Metabolic pathways reported to be significantly altered in the omics-based studies included in this review. Circle size and color intensity is proportional to the number of studies where the metabolic pathway has been found to be altered.

This review summarizes the most relevant findings reported in omics-based studies focused on the characterization of metabolic alterations associated with PD-specific phenotypes. Overall, these studies have revealed that alterations in glycogen, glucose, lipids and aminoacids, nucleotide metabolism and TCA cycle are the most frequently observed (Figure 2). Notably, metabolic changes in glycogen and glucose metabolism are the most representative.

The results from these studies highlight the presence of a deep metabolic remodeling in this disease and confirm the potential of omics-based approaches in lysosomal diseases to reveal clinical and biological associations to generate pathophysiological hypotheses. Also, these studies have demonstrated how some of these metabolic alterations can be reverted following different therapeutic approaches. Different alterations in specific metabolites and metabolic enzymes have been reported in these studies (Table S1). Although the mean-age difference between the groups of samples included in some of these studies together with the unbalanced number of samples included in some groups, usually due to ethical considerations and pediatric recruitment difficulties, represent a drawback in these studies, the significant metabolic differences identified in relation to these pathologies and the response to current treatments may help to characterize pathophysiological mechanisms underlying this disease and shed the light to set future targeted studies.

## 6. Future Perspectives

Due to the high clinical heterogeneity among PD patients, individual phenotyping and patient monitoring are essential for optimal management of these patients. Although great advances have been achieved in recent years towards improving routine testing of many LSDs [137,138,139,140,141,142], further characterization of specific phenotypes that correlate with clinical characteristics of patients (e.g., early/late-onset of disease, presentation of symptoms, etc.) or efficacy of therapies could be very valuable for the management of LSDs patients and for guiding treatment decisions [41]. This would be especially relevant in LSDs such as PD [63], where both diagnosis and treatment at the earliest possible stage is critical for patient prognosis [26,56], and where there is a poor correlation between genotype and clinical manifestations of the disease [15,143,144,145,146].

Future studies including larger cohorts of patients and time-series measurements for treatment monitoring will be needed to verify the biological relevance of the metabolic alterations already reported in PD patients and the following response to treatments. Also, the application of computational-based data augmentation techniques to create synthetic sets of samples that have proven to be an adequate strategy to overcome the limited number of samples often found in other LSDs datasets [147] could be of interest in PD studies. A significant number of more close-to-patients models have been developed in recent years [148,149,150]. The information derived from the metabolomic study of these models could have important implications for assessing these differences and for improving drug targeting and clinical efficacy of PD therapies that are currently under development. Moreover, these approaches could help to promote the development of novel therapies targeting disease mechanisms that are common to other LSDs, similar to autophagy, inflammation and other directed therapies currently under evaluation [151,152,153,154]. Finally, the integration of data from different omics experimental approaches (e.g., genomics, transcriptomics, proteomics and metabolomics) may represent a powerful strategy to parse the genotype–phenotype complexity of PD. Particularly, another experimental approach that has proven its value in characterizing CNS progression [155] and response to therapy [156,157], but also in defining when to start therapy [26] in PD patients, is magnetic resonance imaging (MRI). Hence, the development of new bioinformatics tools for the integration of radiomics and metabolomics data, directed to the characterization of the prognostic profile associated with different subgroups of PD patients, would be of benefit for the identification of specific phenotypes that could be clinically exploited for improving the management of PD patients [158].

## Figures and Tables

**Table 1:** Omics-based studies focused on the identification of metabolic biomarkers for PD diagnosis.

Study	Study Design	Sample	Omics-Based Approach	Major Findings ^†^
Sidorina et al. [99]	13 HC 12 PD	Plasma	nLC-MS/MS SWATH and LC-IMS/MS	nLC-MS/MS SWATH: ↑ LDHB, PKM and ↓ GPLD1 and PON1 LC-IMS/MS: ↑ phosphatidylcholines and ↓ lysophosphatidylcholines
Semeraro et al. [94]	Urine: 75 HC 4 PD	Urine and DUS	UHPLC-MS/MS with MRM	↑ Glc4 and M4
	DUS: 12 HC 2 PD			
de Moraes et al. [100]	21 HC 13 PD	Urine	LC-HRMAS	↑ Glc4, creatine, sorbitol/mannitol, L-phenylalanine, *N*-acetyl-L-aspartic acid and ↓ *N*-acetyl-4-aminobutanal and 2-aminobenzoic acid
Hagemeijer et al. [101]	121 HC 18 PD	Urine	UHPLC/HRAM MS	↑ Glc4, Hex7 and Hex6

DUS: dried urine spots, Glc4: glucose tetrasaccharide, GPLD1: glycosylphosphatidylinositol specific pshospholipase D1, HC: healthy control, LC-HRMAS: liquid chromatography-high resolution mass spectrometry, LC-IMS/MS: liquid chromatography combined with ion mobility mass spectrometry, LDHB: lactate dehydrogenase B, M4: maltotetraose, MRM: multiple reaction monitoring, nLC-MS/MS SWATH: nano-liquid chromatography with tandem mass spectrometry and sequential window acquisition of all theoretical spectra, PD: Pompe disease, PKM: pyruvate kinase M1/2, PON1: paraoxonase 1, UHPLC/HRAM MS: ultra-high performance liquid chromatography with a high-resolution accurate mass mass spectrometry, UHPLC-MS/MS: ultra-high performance liquid chromatography mass spectrometry. ^†^ Direction of variation, considering the healthy group as a reference (↑: higher levels than reference group; ↓: lower levels than reference group).

**Table 2:** Omics-based studies focused on the characterization of specific PD metabolic phenotypes.

Study	Study Design	Sample	Omics-Based Approach	Major Findings ^†^
Lim et al. [84]	10 WT 14 GAA-KO	Muscle tissue	CE-MS	↑ histidine, lysine, threonine, alanine, aspartate, glutamine and serine
Meena et al. [112]	6 WT 6 GAA-KO	Muscle tissue	CE-TOF/MS and CE-QqQMS	↑ Gal1P, UDP-glucose, acetyl-CoA, citrate, succinate, fumarate, malate, carnitine, and ↓ G1P, G6P, F6P, F1,6B, pyruvate and lactate
Kinton et al. [110]	10 HC 8 LOPD	Muscle tissue	Transcriptome profiling	↑ lysosomal function, glycolysis, lipid metabolism and calcium homeostasis, and ↓ mitophagy pathway
Moriggi et al. [111]	15 HC 10 LOPD	Muscle tissue	2D-DIGE and LC-MS/MS	↑ glycolysis and ↓ OXPHOS
de Moraes et al. [100]	8 IOPD 14 LOPD	Urine	LC-HRMAS	↓ Glc4, Hex5, Hex6, and Hex7

2D-DIGE: two-dimensional difference gel electrophoresis, CE-MS: capillary electrophoresis-mass spectrometry, CE-QqQMS: capillary electrophoresis-triple quadrupole mass spectrometry, CE-TOF/MS: capillary electrophoresis-time of flight mass spectrometer, F1,6B: fructose-1,6-biphosphate, F6P: fructose-6-phosphate, G1P: glucose-1-phosphate, G6P: glucose-6-phosphate, GAA: acid alpha-glucoside, Gal1P: galactose 1-phosphate, Glc4: glucose tetrasaccharide, HC: healthy control, IOPD: infantile-onset Pompe disease, KO: knockout, LC-MS/MS: liquid chromatography-mass spectrometry, LOPD: late-onset Pompe disease, OXPHOS: oxidative phosphorylation. ^†^ Direction of variation, considering the first group as a reference (↑: higher levels than reference group; ↓: lower levels than reference group).

**Table 3:** Omics-based studies focused on the characterization of the metabolic response to different therapeutic interventions under evaluation for the treatment of PD.

Study	Study Design	Treatment	Sample	Omics-Based Approach	Major Findings ^†^
Moriggi et al. [111]	10 LOPD untreated 10 LOPD treated	ERT	Muscle tissue	LC-MS/MS	↓ glycolysis and gluconeogenesis
Kinton et al. [110]	8 LOPD untreated 8 LOPD treated	ERT	Muscle tissue	Transcriptome profiling	↑ mitophagy and ↓ sphingolipid and phospholipid metabolism, cytosolic calcium
Meena et al. [121]	6 untreated GAA-KO 6 treated GAA-KO	ERT	Muscle tissue	CE-TOF/MS and CE-QqQMS	↓ Gal1P, UDP-glucose, acetyl-CoA, citrate, succinate, fumarate, malate, and ↑ G1P, G6P, F6P, pyruvate and lactate
Colella et al. [128]	3 untreated GAA-KO 3 treated GAA-KO	AAV	Muscle tissue and spinal cord	Transcriptome profiling	In skeletal muscle: ↑ glycogen degradation, glucose and glucose-1-phosphate degradation, and serine and glycine biosynthesis In spinal cord: ↑ nervous system disease, neuroinflammation, immunity and energy sensing

AAV: adeno-associated virus, CE-QqQMS: capillary electrophoresis-triple quadrupole mass spectrometry, CE-TOF/MS: capillary electrophoresis-time of flight mass spectrometer, ERT: enzyme replacement therapy, F6P: fructose-6-phosphate, G1P: glucose-1-phosphate, G6P: glucose-6-phosphate, GAA: acid alpha-glucoside, Gal1P: galactose 1-phosphate, KO: knockout, LC-MS/MS: liquid chromatography-mass spectrometry, LOPD: late-onset Pompe disease. ^†^ Direction of variation, considering the first as a reference (↑: higher levels than reference group; ↓: lower levels than reference group).

## Data Availability

Not applicable.

## Supplementary Materials

The following supporting information can be downloaded at: https://www.mdpi.com/article/10.3390/biology12091159/s1, Figure S1. Flow diagram of the systematic search followed for the selection of the studies included in the review; Table S1. Metabolites and metabolic enzymes reported to be significantly altered in the studies included in this review.
