# Supplementary material for: Omics-Based Approaches for the Characterization of Pompe Disease Metabolic Phenotypes
Source: Biology (Basel). 2023 Aug 23;12(9):1159. doi: 10.3390/biology12091159 (PMC10525434; doi:10.3390/biology12091159)
Supplement: Supplementary file 1 [file biology-12-01159-s001.zip › PD_biology_SupplementaryMethods.pdf]

Supplementary methods

Literature search

A systematic search was conducted on 15th May 2023, on PubMed, using the following terms: ("pompe" or "acid alpha- glucosidase" or "acid alpha glucosidase" or "pompe disease" OR "glycogen storage") AND ("omics" OR "transcriptomics" OR "proteomics" OR "genomics" OR "epigenomics" OR "lipidomics" OR "metabolomics" OR "omics-based" OR "metabolic phenotype" or "metabolome" OR "microbiome" OR "genome" or "proteome" or "epigenome" or "lipidome" or "transcriptome" or "NMR" OR "LC-MS" OR "LC-MS/MS" OR "UPLC" OR "UHPLC") NOT review. This search resulted in a total of 410 articles (**Figure S1**). Only original articles written in English were considered for the inclusion in this review. Then, article titles were examined to evaluate their eligibility according to their relevance on the aim of this review. Thus, only those that could be focused on metabolic phenotyping and related to Pompe disease or LSDs were retained, leading to 209 publications. Next, abstracts of full-text publications were examined to select studies where omics-based approaches were applied to the characterization of PD or LSDs metabolic phenotypes, resulting in a total of 56 articles. Finally, full-text articles were thorough inspected to confirm their association with PD and the application of omics technologies to characterize the metabolic profile of biological samples. This resulted in the final selection of 9 publication. Different informative data were extracted from this final subset of publications, including methodology, experimental design, sample type, metabolites and/or metabolic enzymes, etc.

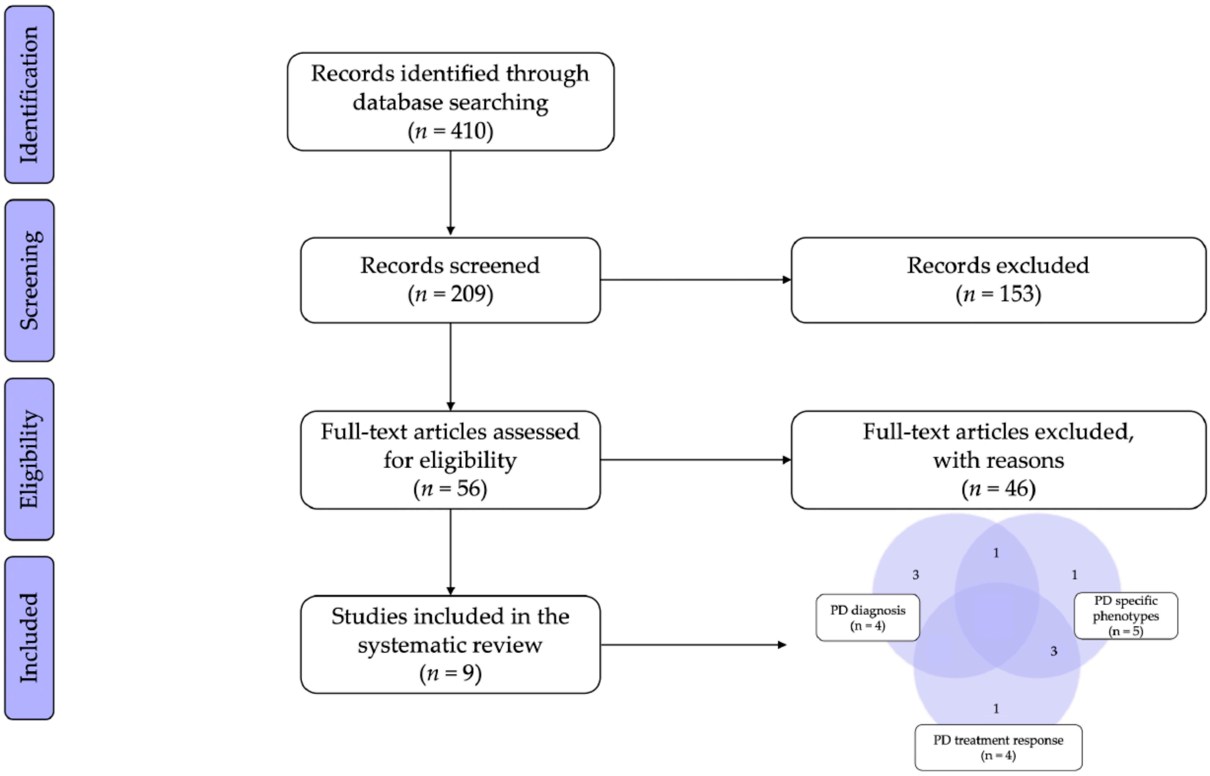

Figure S1. Flow diagram of the systematic search followed for the selection of the studies included in the review
